# The effect of a 4-week, remotely administered, post-exercise passive leg heating intervention on determinants of endurance performance

**DOI:** 10.1007/s00421-024-05558-4

**Published:** 2024-07-25

**Authors:** Kevin John, Joe Page, Shane M. Heffernan, Gillian E. Conway, Neil E. Bezodis, Liam P. Kilduff, Brad Clark, Julien D. Périard, Mark Waldron

**Affiliations:** 1 Research Institute for Sport and Exercise, University of Canberra, Canberra, Australia; 2 Applied Sports Science Technology and Medicine (A-STEM) Research Centre, Faculty of Science & Engineering, Swansea University, Bay Campus, Swansea, SA1 8EN Wales, UK; 3 Institute of Life Science, Faculty of Medicine, Health and Life Sciences, Swansea University, Swansea, UK; 4 Welsh Institute of Performance Science, Swansea University, Swansea, UK; 5 School of Health and Behavioural Sciences, University of the Sunshine Coast, Maroochydore, QLD Australia

**Keywords:** Post-exercise, Passive leg heating, Temperate, Endurance performance

## Abstract

**Purpose:**

Post-exercise passive heating has been reported to augment adaptations associated with endurance training. The current study evaluated the effect of a 4-week remotely administered, post-exercise passive leg heating protocol, using an electrically heated layering ensemble, on determinants of endurance performance.

**Methods:**

Thirty recreationally trained participants were randomly allocated to either a post-exercise passive leg heating (PAH, *n* = 16) or unsupervised training only control group (CON, *n* = 14). The PAH group wore the passive heating ensemble for 90–120 min/day, completing a total of 20 (16 post-exercise and 4 stand-alone leg heating) sessions across 4 weeks. Whole-body (peak oxygen uptake, gas exchange threshold, gross efficiency and pulmonary oxygen uptake kinetics), single-leg exercise (critical torque and NIRS-derived muscle oxygenation), resting vascular characteristics (flow-mediated dilation) and angiogenic blood measures (nitrate, vascular endothelial growth factor and hypoxia inducible factor 1−α) were recorded to characterize the endurance phenotype. All measures were assessed before (PRE), at 2 weeks (MID) and after (POST) the intervention.

**Results:**

There was no effect of the intervention on test of whole-body endurance capacity, vascular function or blood markers (*p* > 0.05). However, oxygen kinetics were adversely affected by PAH, denoted by a slowing of the phase II time constant; τ (*p* = 0.02). Furthermore, critical torque–deoxygenation ratio was improved in CON relative to PAH (*p* = 0.03).

**Conclusion:**

We have demonstrated that PAH had no ergogenic benefit but instead elicited some unfavourable effects on sub-maximal exercise characteristics in recreationally trained individuals.

**Supplementary Information:**

The online version contains supplementary material available at 10.1007/s00421-024-05558-4.

## Introduction

Whole-body passive heating has gained attention as a strategy for enhancing the adaptative stimulus created by exercise. Indeed, when applied in post-exercise formats, whole-body passive heating has been reported to enhance aerobic capacity more than exercise only controls, among athletes of recreational to elite status (Dalleck et al. 2019; Kirby et al. 2021; Scoon et al. 2007. Given that whole-body heating modalities are systemically thermogenic and, therefore also capable of inducing heat acclimation (Racinais et al. 2017; Zurawlew et al. 2016), it is conceivable that the reported endurance adaptations are explained by enhancement of central-O_2_ transport (i.e. plasma volume and haemoglobin mass), as this has been associated with repeated whole-body hyperthermia (Périard et al. 2015). An inherent limitation of using whole-body passive heating to optimise training is the dependence upon laboratory visitation or requirement of specialist equipment. This poses a logistical burden, especially in a post-exercise format, and it may also be difficult to tolerate immersive whole-body strategies at the suggested dosages (Steward et al. 2023). For these reasons, accessibility and adherence to a passive heating programme for recreationally active or trained populations might be restricted in real-world settings. Furthermore, it is unclear whether modalities that only increase local tissue temperature are similarly ergogenic for individuals regularly partaking in endurance training.

Two major observations may support the abovementioned notion. First, acute lower leg heating can result in threefold increases leg-arterial blood flow and shear rate (Chiesa et al. 2016), which is also accompanied by a greater expression of angiogenic factors (endothelial nitric oxide synthase: eNOS, vascular endothelial growth factors: VEGF) within skeletal muscles post-heating (Gibson et al. 2023; Kuhlenhoelter et al. 2016). Second, chronic local heating has been reported to improve metabolic and contractile properties of the musculature. For instance, Kim et al. (2020) reported that lower leg hot water circulating suits applied over 8 weeks increased eNOS expression and capillary contacts around type II fibres. Furthermore, 6 consecutive days of short-wave diathermy increased coupled and uncoupled mitochondrial respiratory capacity (Hafen et al. 2018). In addition to improvements in cellular respiratory function, protein content of complex I, V and peroxisome-activated receptor-gamma coactivator was also increased, indicating mitochondrial biogenesis (Hafen et al. 2018). Finally, some have reported increases in cross-sectional area of muscle exposed to local heating (Goto et al. 2007, 2011), but more importantly, improvements in torque production and fatigue resistance have been observed with repeated lower limb heating (Goto et al. 2007; Kim et al. 2019, 2020). As such, local heating modalities may favour peripheral adaptations that improve O_2_ availability and metabolism, which significantly contributes to the ‘in-series system’ determining aerobic capacity (Wagner 2011).

Recent advances in clothing technology, such as electrically heated garments, might provide a more mobile local passive heating strategy (Wang et al. 2010). These garments have been used in sporting events to offset post-exercise decay of muscle temperature (Faulkner et al. 2013; McGowan et al. 2017) and, with sufficient insulative adjustments, might provide the stimulus necessary for sustained physiological adaptation. To our knowledge, no study to date has used electrically heated garments as part of a remote (i.e. home-based) passive heating system, to augment endurance adaptations. Therefore, the aims of the study were to (i) compare the effects of a 4-week lower body, post-exercise passive heating intervention, using an electrically heated layering ensemble alongside routine recreational exercise, versus exercise-only controls on markers of endurance performance; peak aerobic capacity ($\mathop {\text{V}}\limits^{.}$ O_2peak_), pulmonary oxygen kinetics ($\mathop {\text{V}}\limits^{.}$˙O_2_ kinetics), gross-efficiency, gas exchange threshold (GET), critical torque (CT) and skeletal muscle oxygenation, among recreationally trained individuals in temperate conditions; and (ii) determine the systemic adaptations related to passive heating by monitoring changes in brachial artery vascular function (flow mediated dilation) and the expression of key angiogenic blood markers (i.e. nitric oxide metabolites, hypoxia-inducible factor 1 alpha: HIF1-α and VEGF). We hypothesised that supplementing routine endurance training with passive heating intervention will enhance endurance capacity compared to control training and that this would be linked to changes in vascular function and systemic angiogenesis.

## Materials and methods

### Participants and ethical approval

A total of 34 participants volunteered to take part in the study. Participants were randomly assigned to the post-exercise passive leg heating (PAH) or control (CON) group, accounting for baseline fitness and training volume (see group allocation). An *a-*priori sample size was calculated using G*Power (version 3.1, Universität Düsseldorf, Germany) based on reported changes in $\mathop {\text{V}}\limits^{.}$O_2peak_ after whole-body PAH (*F* = 0.575; (Dalleck et al. 2019)). Fifteen participants per group provided a power of 0.80 and *α* = 0.05 using an ANCOVA model with numerator degrees of freedom = 1. To account for attrition, the study over-recruited by two participants per group. Accordingly, there was 1 drop-out in PAH and 3 drop-outs in CON, leading to a final sample size of *n* = 16 (3 females) in PAH and *n* = 14 (2 females) in CON. The female cohort included *n* = 3; self-reported eumenorrheic cycle who were not using any hormonal contraceptives, *n* = 1; intrauterine hormone coil user, and *n* = 1; monophasic oral contraceptive user. Prior to recruitment, participants were screened for inclusion criteria, which were: (i) taking part in regular endurance training (> 150 min/week), (ii) not affected by any cardiovascular or neuromuscular pathologies, and (iii) have not taken part in any structured training in the heat in the past year or visited a tropical country in the previous three months. The study recruited participants from various endurance training disciplines which included triathlon (*n* = 8), cross-country running (*n* = 6), road running (*n* = 5), cycling (*n* = 3), football (*n* = 3), netball (*n* = 3) and squash (*n* = 2). Participant characteristics, baseline $\mathop {\text{V}}\limits^{.}$˙O_2peak_ and training volume are listed in Table 1. The study was conducted in the UK, between the months of January and July (temperature ranging from ~ 10 to 21 °C). This timeline ensured that all participants were unacclimatised to the heat. All participants were asked to avoid using hot baths and saunas throughout the participation period (four weeks) of the study. The study was approved by the Swansea University, College of Engineering Research Ethics and Governance Committee (KJ_01-10-21) and all participants provided written informed consent. This study was conducted in accordance with the Declaration of Helsinki agreement (2018).

**Table 1:** Baseline participants characteristics and intervention training characteristics for passive heating (*n* = 16) and control (*n* = 14)

	Passive Heating		Control	
Age (years)	25	± 6	30	± 7
Body mass (kg)	72.46	± 13.64	72.64	± 9.70
Stature (cm)	176.38	± 7.82	177.00	± 8.32
$\mathop {\text{V}}\limits^{.}$O_2peak_ (L/min)	3.27	± 0.72	3.65	± 0.78
TV (min)	1618	± 606	1834	± 1059
ED sessions	17	± 3	15	± 3
ED volume (min)	1197	± 380	1253	± 584
RS sessions	3	± 2	3	± 2
RS volume (min)	149	± 103	108	± 60
TL1 (min × RPE)	3220	± 1258	3540	± 2276
TL2 (min × RPE)	3092	± 1323	3279	± 1819

*TL* training load, *TL1* TL between PRE to MID, *TL2* TL between MID to POST, $\mathop V\limits^{.}$*O*_*2peak*_ maximal oxygen uptake, *TV* baseline training volume (4 week estimate), *ED* endurance training, *RS* resistance training

### Study overview

After the initial screening, a familiarisation visit was organised for each participant, 1 week prior to starting the study, which included physical participation in reduced formats of all tests and estimating their projected training volume for the proceeding 4 week block.

**Fig. 1:**
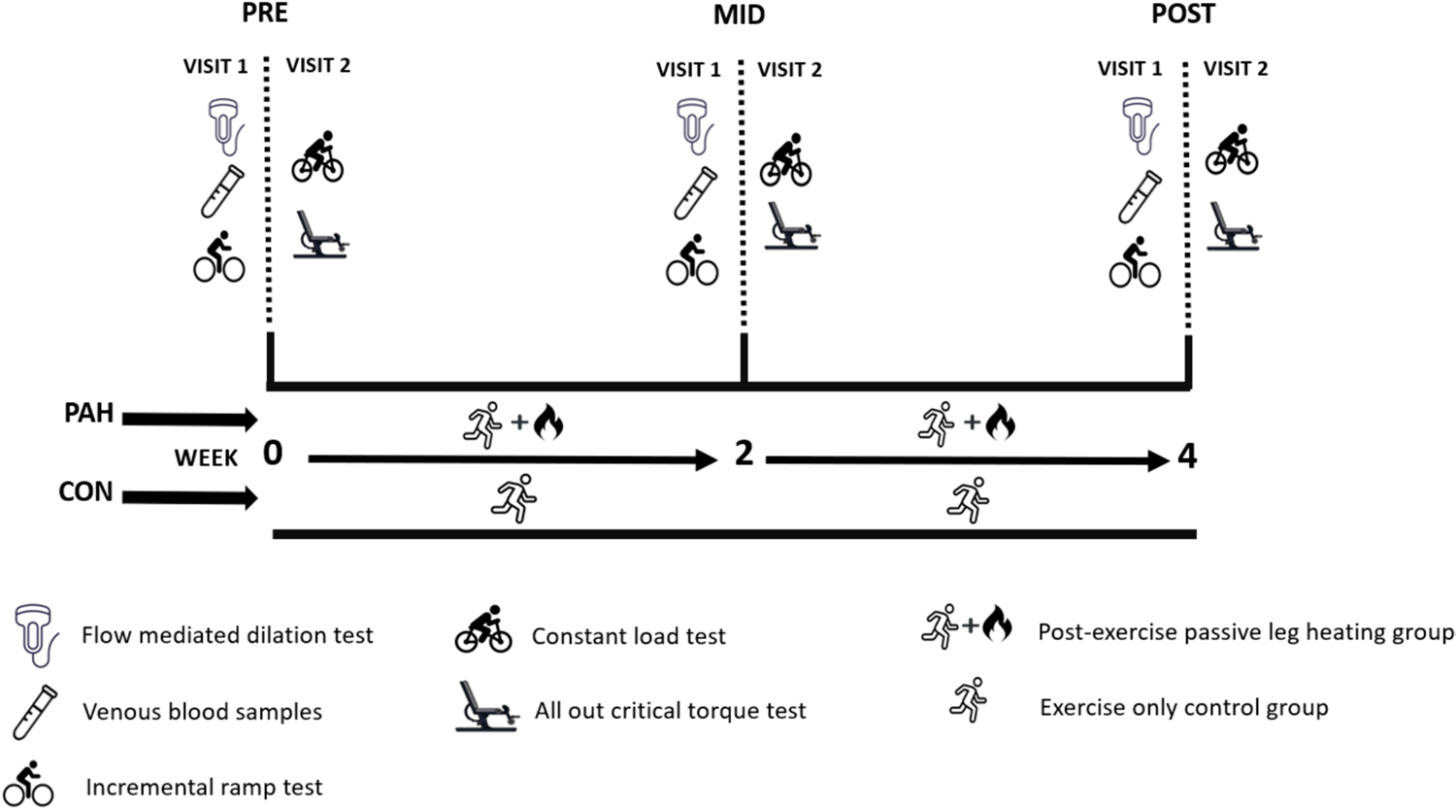
Schematic representation of the study timeline. The PAH group were required to complete five sessions of passive leg heating per week out which a minimum of three session had to be completed in a post-exercise format, whereas the CON group continued normal endurance training

Following this, participants reported to the laboratory on six different occasions across 4 weeks. Data collection was separated into three testing ‘blocks’ (Fig. 1): 2 consecutive days in the 72 h prior to the first day of intervention (PRE); 2 consecutive days in the middle of the 4 week intervention after a 24 h recovery and following ten sessions of leg heating or CON (MID); 2 consecutive days after a 24 h recovery following the final ten leg heating sessions or CON (POST) of the 4 week intervention. Visit 1 required participants to report to the laboratory in a fasted state for arterial imaging, after which venous blood samples were collected. Participants were then given a 60–120 min break to consume food of their choice (recorded and standardised for all subsequent visits), and on return, completed a ramp protocol test to establish $\mathop {\text{V}}\limits^{.}$O_2peak_. On Visit 2, participants completed a multiple-bout constant load test on a cycle ergometer to determine rest-to-exercise $\mathop {\text{V}}\limits^{.}$˙O_2_ kinetics, followed by a single-leg critical torque test on an isokinetic dynamometer. During the 4-week study period, participants in both groups were required to complete a minimum of 12 endurance training sessions (3 per week) and were requested to keep their training regimen consistent with their previous (typical, pre-intervention) training. At pre-screening, it was ensured that the training of participants at the beginning of the intervention did not coincide with a planned overload in intensity or volume, which was monitored using an online log for the remainder of the study. Training load for each session was calculated by multiplication of training duration (min) and the 0–10 rating of perceived exertion (RPE) (Foster et al. 2001), which is a valid metric for estimating internal training load (Mann et al. 2019; Seiler and Kjerland 2006).

The order of testing remained consistent for each testing block and participant throughout the study. Participants were asked to refrain from strenuous exercise, caffeine or use of any other dietary supplements and alcohol in the 24 h prior to and during each testing block. Additionally, participants were required to replicate the same dietary pattern 24 h prior to and during each testing block. All testing took place between 7:00 and 10:00 am in temperate conditions (~ 20 ℃) with participants required to wear the same t-shirt, shorts and training shoes for all visits.

### Procedures

#### Group allocation

The randomisation of group allocation was performed using a Microsoft Excel spreadsheet (MinimizeMeansAsRecruit.xls) developed by Hopkins (2010). This approach allocated participants to groups as the study progressed, based on minimisation of the standardised between-group differences in (i) baseline cardiorespiratory fitness (indicated by $\mathop {\text{V}}\limits^{.}$O_2peak_ measured PRE) and (ii) 4-week training volume (in min) estimated during the familiarisation visit.

#### Determination of $\mathop {\text{V}}\limits^{.}$O_2peak_ and GET

An incremental ramp test was used to determine $\mathop {\text{V}}\limits^{.}$O_2peak_ using an electronically braked cycle ergometer (Lode, Excalibur sport 06, Groningen, Netherlands). Participants first completed a 5 min warm-up cycling at 50 W with a self-selected cadence. Following 3 min of recovery, the ramp test to volitional exhaustion was conducted, starting at 10 W and progressing by 25 W/min. Participants were instructed to maintain a cadence of 70–80 rev/min for the duration of the test. All pulmonary gas measurements ($\mathop {\text{V}}\limits^{.}$O_2_, carbon dioxide production; $\mathop {\text{V}}\limits^{.}$CO_2_, minute ventilation; $\mathop {\text{V}}\limits^{.}$E, and respiratory exchange ratio; RER) were recorded using a breath-by-breath gas analyser (Vyntus CPX, Carefusion, Hoechberg, Germany 234 GmbH). Prior to testing the gas analyser was calibrated using known gas concentrations (15.94% O_2_, 5.00% CO_2_). The turbine transducer was volume-calibrated automatically by the system, using flow rates 2 L/s and 0.2 L/s. Criteria used for test cessation and achieving $\mathop {\text{V}}\limits^{.}$O_2peak_ were: (i) reaching volitional exhaustion, (ii) inability to maintain a cadence > 70 rev/min, and (iii) an RER > 1.15. The highest mean $\mathop {\text{V}}\limits^{.}$O_2_ value achieved across 30 s of the test was recorded as $\mathop {\text{V}}\limits^{.}$O_2peak_ and peak power output was recorded as the power output achieved at test cessation subtracted by 2/3rd of the ramp rate (16.67 W/min) (Whipp et al. 1981).

The GET was determined using the simplified v-slope method (Schneider et al. 1993) and previous recommendations (Wasserman 1984). The criteria used for visual detection of the GET were: (i) the first point of deviation in linearity between the plots of $\mathop {\text{V}}\limits^{.}$CO_2_ and $\mathop {\text{V}}\limits^{.}$O_2_, and (ii) the first rise in the ventilatory equivalent for O_2_ ($\mathop {\text{V}}\limits^{.}$E/$\mathop {\text{V}}\limits^{.}$O_2_) without any simultaneous increase in the ventilatory equivalent of CO_2_ ($\mathop {\text{V}}\limits^{.}$E/$\mathop {\text{V}}\limits^{.}$CO_2_). Agreement between two independent assessors was required to confirm the identification of the GET.

#### Determination of $\mathop {\text{V}}\limits^{.}$O_2_ kinetics

Participants performed a series of three square-wave bouts from unloaded-to-loaded cycling on the same cycle ergometer. The duration for each bout was 11 min, beginning with a 3 min resting period, sitting stationary on the ergometer, followed by 3 min of unloaded (~ 0 W) pedalling, which was proceeded by an instantaneous transition of pedalling for 5 min against a constant load. The load selected (power output) was aimed to elicit 90% of the $\mathop {\text{V}}\limits^{.}$O_2_ determined at GET and was adjusted after each testing block depending on changes in GET. Participants were required to maintain a steady cadence of 70–80 rev/min between all exercise transitions. Breath-by-breath pulmonary gas data were collected simultaneously using the same metabolic cart described above.

Breath-by-breath $\mathop {\text{V}}\limits^{.}$O_2_ data acquired from the constant load (i.e. mechanical load) test was first corrected for errant breaths and all breaths that lay ± 3 standard deviations (SD) away from the local mean were removed from the data series (Lamarra et al. 1987). $\mathop {\text{V}}\limits^{.}$O_2_ data for each rest-to-exercise transition was then linearly interpolated to 1 s intervals, time aligned, and ensemble averaged at each 1 s interval to produce a single rest-to-exercise $\mathop {\text{V}}\limits^{.}$O_2_ response. The final data were then averaged at 5 s intervals (Keir et al. 2010) and the $\mathop {\text{V}}\limits^{.}$O_2_ response after baseline was modelled using a mono-exponential function (Benson et al. 2017; Lamarra et al. 1987; Rossiter et al. 2002):

1 $$\mathop {\text{V}}\limits^{.} {\text{O}}_{{2({\text{t}})}} = \mathop {\text{V}}\limits^{.} {\text{O}}_{{2{\text{BASE}}}} + \mathop {\text{V}}\limits^{.} {\text{O}}_{{2{\text{AMP}}}} [1 - {\text{e}}^{{ - ({\text{t}} - {\text{TD}})/\tau )}} ],$$

where $\mathop {\text{V}}\limits^{.}$O_2_ at any given time point *t* is equal to the sum of $\mathop {\text{V}}\limits^{.}$O_2_ during unloaded cycling ($\mathop {\text{V}}\limits^{.}$O_2BASE_ [mL]) and the exponential function comprising an amplitude ($\mathop {\text{V}}\limits^{.} {\text{O}}_{{{\text{2AMP}}}}$ [mL]: highest steady-state $\mathop {\text{V}}\limits^{.}$O_2_ achieved during loaded exercise above $\mathop {\text{V}}\limits^{.}$O_2BASE_), time constant (τ [s]) and a time delay (TD [s]). Here, τ represents the time required to attain 63% of the total amplitude ($\mathop {\text{V}}\limits^{.} {\text{O}}_{{{\text{2AMP}}}}$) after an initial TD.

The first 20 s of the unloaded-to-loaded exercise transition was defined as the cardio-dynamic phase I response (Whipp et al. 1982) and was removed prior to fitting the model. This was done to ensure that the model only characterised the phase II exponential response that is related to muscle O_2_ consumption and subsequent achievement of steady-state (Rossiter et al. 1999). The parameters of $\mathop {\text{V}}\limits^{.}$O_2_ kinetics: $\mathop {\text{V}}\limits^{.} {\text{O}}_{{{\text{2AMP}}}}$, τ & TD were determined using non-linear least squares regression, estimated from the line of best-fit. The fitting window was set to 240 s of the phase II step-transition data. The above data handling and processing procedure was completed using the open-source code (https://github.com/fmmattioni/whippr) developed on R framework and was consistent for all participants.

#### Calculation of gross efficiency

The $\mathop {\text{V}}\limits^{.}$˙O_2_ and RER values from the final 120 s (constant load) of the final breath-by-breath data developed for $\mathop {\text{V}}\limits^{.}$O_2_ kinetics analysis (explained above) was averaged and gross efficiency was determined using the equations (Garby and Astrup 1987):

2 $${\text{Gross efficiency }} = {\text{ Mechanical power output/metabolic input}},$$

3 $${\text{Metabolic~input~}} = {\text{~}}\left( {\mathop {\text{V}}\limits^{.} {\text{O}}_{{\text{2}}} } \right){\text{~}} \times {\text{~}}\left[ {\left( {4940{\text{~}} \times {\text{RER}}} \right) + 16040} \right]/60.$$

The mechanical power output (W) was taken as the sub-maximal load used (90% of GET) during the constant work rate test. $\mathop {\text{V}}\limits^{.}$O_2_ in Eq. 3 was measured in L/min and represented the new steady-state achieved during the loaded phase of the test. Furthermore, due to the sub-maximal nature of the test, RER values were always below 1.0, therefore no further corrections were necessary (Noordhof et al. 2010).

#### Determination of CT

CT was determined using a single-leg isometric 5 min all out critical test (5 min AOCT) developed by Burnley (2009). All single-leg assessments were performed on the right leg, with participants seated in the chair of an isokinetic dynamometer (HUMAC NORM™, Cybex, Massachusetts, USA). Following system warm-up and calibration, the distal tibial region (above the distal tibiofibular joint) was secured using a Velcro strap to the padded end of an extended-lever arm attached to the dynamometer. The chair was positioned such that the lateral epicondyle of the right femur was perpendicular to the axis of rotation of the lever arm. Participants sat with their back rested on the chair ensuring the hip was placed at a 90° angle and the knee held at a 75° angle (complete knee extension defined as the anatomical 0^°^) for the complete duration of the test. Both shoulders and waist were strapped securely to the chair and participants were asked to hold the fixed side handles when performing isometric muscle contractions. The final seating position was recorded during the familiarisation visit and remained constant for all subsequent test visits.

Following a sub-maximal isometric warm-up, participants performed three maximal voluntary contractions (MVC), forcefully contracting the knee extensors for 3 s with a 60 s rest in between. MVC was defined as the peak torque (N.m) produced between the three contractions. After establishing MVC, participants were given a 3 min rest period during which they were made aware of their MVC and instructed to match the score during the 5 min AOCT. Participants were also informed to expect extreme feelings of fatigue after the initial contractions but were instructed to produce maximal efforts for the entire duration of the test. The 5 min AOCT consisted of 60 consecutive contractions, with each contraction cycle consisting of a 3 s MVC followed by 2 s of rest (Burnley 2009). To prevent a reserve pacing strategy, participants were blinded to the duration or the number of contractions remaining. The test was conducted with verbal cues of “go” and “relax” for the contraction and relaxation phase, respectively, and participants were aided by a visual force trace. The dynamometer was set at a sampling frequency of 100 Hz.

The raw torque data (T) were analysed using a code implemented in Python (version 3.10.8) where mean torque was calculated for each 3 s contraction cycle and impulse (N.m.s) was calculated as the area under the torque-time curve. The mean torque of the last six contractions was averaged to establish critical torque (CT), which represented the last 30 s of the 5 min AOCT (Burnley 2009). Total impulse (I) was taken as the sum of the impulse calculated for each 3 s contraction cycle. The work done above CT, defined as impulse prime (I′) was determined by a modification of the two-parameter model proposed by Monod and Scherrer (1965).

4 $${\text{I}}^{\prime } {\text{ = I }}{ - }{\text{ CT }} \times {\text{ Tlim}}{.}$$

where Tlim represents the duration of the all-out test in seconds (i.e. 300 s).

#### Measurement of muscle oxygenation

Muscle oxygenation was monitored simultaneously during the 5 min AOCT using near-infrared spectroscopy (NIRS). This device works on the differential light absorption properties of haemoglobin (Hb) and myoglobin (Mb) in the presence (oxyhaemoglobin: O_2_Hb) or absence (deoxyhaemoglobin: HHb) of oxygen (Hamaoka et al. 2007). The study used a continuous-wave NIRS unit (PortaMan, Artinis medical systems, Amsterdam, Netherlands) to record relative changes in concentrations of Δ[O_2_Hb], $\Delta \left[ {{\text{HHb}}} \right]$ (arbitrary units; a.u.) and estimate tissue saturation index (TSI) of the quadriceps during the 5 min AOCT test. TSI is calculated as a ratio of Δ[O_2_Hb] to total [Hb] (Δ [O_2_Hb] +  Δ[HHb]) and expressed as a percentage (Hamaoka et al. 2007). The NIRS unit was placed longitudinally over the right vastus lateralis muscle, at ~ 50% of the thigh length measured between the greater trochanter and lateral epicondyle of the femur. The site of unit placement was prepared prior to attachment by removing any hair (dry shave) and cleaned using alcohol swabs. The unit was secured to the region of interest using an elasticated bandage and then fastened with micropore tape to prevent unit-movement artefacts during contractions. Lastly, the attached unit was covered with a black indelible cloth, taped on the anterior portion of the thigh to prevent external light from interfering with the NIRS signal. A permanent marker was used to mark the area of placement at PRE, to ensure repeatable placements at MID and POST. The NIRS unit used a dual-wavelength of 760 and 850 nm, with source-detector spacing of 30, 35 and 40 mm. Before initiating the isometric test, the unit was connected to a personal computer via Bluetooth technology and data were acquired in real-time through the OxySoft software (Artinis medical systems, Amsterdam, Netherlands) at a sampling frequency of 10 Hz.

Similar to analysis of raw torque (T) data (5 min AOCT), mean TSI and HHb were calculated for each 3 s contraction. Following this, mean torque for every contraction (*n* = 60) was expressed as percentage of the MVC. Here, MVC was taken as the highest mean torque output achieved during the 5 min AOCT. This was repeated for the mean TSI and MVC. Here, MVC was taken [HHb] signal and maximum mean TSI and $\Delta \left[ {{\text{HHb}}} \right]$ were used in place of MVC. Once all the values were expressed as a percentage to their respective maximum, ratios were developed between T%: TSI% (representing mechanical work for a given O_2_ availability) and T%: Δ[HHb]% (representing mechanical work for a given O_2_ extraction). We iteratively determined that the ratio data followed an exponential decay across time (see Fig. 6), which was modelled and characterised accordingly (GraphPad 9.0 Software, San Diego, CA, United States):

5 $${\text{R}}_{{\text{T/TSI}}} {\text{ = CR }}_{{\text{T/TSI}}} { + }\left( {{\text{0R}}_{{\text{T/TSI}}} {\text{ - CR }}_{{\text{T/TSI}}} } \right){ } \times {\text{e}}^{{{\text{( - (X)/}}\tau {)}}} {,}$$

6 $${\text{R}}_{{{\text{T/}}\vartriangle {\text{[HHb]}}}} {\text{ = CR }}_{{{\text{T/}}\vartriangle {\text{[HHb] }}}} {\text{ + (0R }}_{{{\text{T/}}\vartriangle {\text{[HHb]}}}} {\text{ - CR }}_{{{\text{T/}}\vartriangle {\text{[HHb]}}}} { ) } \times {\text{ e}}^{{{\text{( - (X)/ }}\tau {)}}} .$$

Where:

1. R_T/TSI_ and R ${\text{T/}}\Delta {\text{[HHb]}}$ are the ratios at time point X (s) derived from TSI and HHb signal, respectively,
2. 0R_T/TSI_ and 0R ${\text{T/}}\Delta {\text{[HHb]}}$ are the ratios when time point X = 0 s,
3. τ is the time constant expressed in the same unit as X.
4. CR_T/TSI_ and CR ${\text{T/}}\Delta {\text{[HHb]}}$ are the model predicted critical ratios and represent the interaction of CT with oxygenation and deoxygenation.

#### Assessment of brachial artery functioning

A flow mediated dilation test was conducted, according to recent guidelines (Thijssen et al. 2019), to non-invasively assess endothelial-dependent function of the brachial artery. A high-resolution ultrasound device (Esaote, Mylab9, Genova, Italy), equipped with a 10 MHz ultrasound probe, was used for simultaneous measurement of brachial artery diameter and blood flow using the B-mode echo and pulse-wave Doppler velocity, respectively. Real-time changes in the arterial diameter were monitored using a specialised edge-detection software programme (Cardiovascular suite, version 4.3.0), recording at 1 s intervals. On arrival at the laboratory, participants rested in supine position in a quiet, dark and temperature-controlled room for 10 min prior to analysis. An ultrasound probe was placed longitudinally on the brachial artery, proximal to the antecubital fossa with a pressure cuff (Hokanson SC5 Vascular Tourniquet) wrapped distal to it (3–5 cm) on the forearm. Probe placement was recorded (distance from the medial epicondyle) at PRE and repeated for subsequent visits. Baseline artery diameter and blood flow was measured for 1 min, after which the pressure cuff was manually inflated to 220–230 mmHg for 5 min (occlusion period). Post-deflation, diameter and blood flow was measured for another 4 min to capture the complete hyperaemic response following occlusion. All testing was carried out in the morning following an overnight fast and time of analysis was kept constant between visits (± 30 min).

Flow-mediated dilation was calculated as the difference between maximum diameter (D_MAX_ [mm]) achieved post-occlusion and baseline diameter (D_BASELINE_ [mm]) and represented as a %:

7 $${\text{Flow mediated dilation \% = [(D}}_{{{\text{MAX}}}} {\text{ {-} D}}_{{{\text{BASELINE}}}} {\text{)/ D}}_{{{\text{BASELINE}}}} {] } \times { 100}{\text{.}}$$

Shear rate (SR [1/s]) was calculated using the Doppler flow velocity waveform:

8 $${\text{SR = (4 }} \times {\text{ V)/D,}}$$

where V and D represent mean velocity (mm/s) and arterial diameter (mm) at a particular time frame, respectively. In addition to this, SR area under the curve (SR_AUC_), which represents maximal vasodilation area, was calculated from the beginning of vasodilation (post-occlusion) until D_MAX_ using the trapezoidal rule (Thijssen et al. 2019).

#### Blood sampling

Resting venous blood samples (2 $\times$ 10 mL) were drawn from an antecubital vein into 10 mL serum vacutainers. Samples were allowed to coagulate for 20 min at room temperature, after which they were placed on ice for a further 20 min. Samples were centrifuged at 2800*g* for 10 min at 4 °C before being aliquoted into 1.5 mL tubes. All samples were stored at −80 °C for later analysis.

Nitrate concentrations were calculated via the enzyme nitrate reductase assay (R&D Systems, Europe Ltd., Abingdon, UK). Serum samples were filtered (10,000 molecular weight, Amicon™, Merck, Darmstadt, Germany) and twofold diluted prior to assay. Optical density (OD) was determined at 540 nm (wavelength correction at 690 nm), the minimum detectable dose was 0.25 μmol/L. VEGF was determined by ELISA assay (Enzo Life Sciences, Inc. Lausen, Switzerland). Serum samples were diluted prior to assay (1:16) and OD was determined at 450 nm, the limit of detection was 4.71 pg/mL. HIF1-α was measured by Invitrogen assay (Bender Medsystems, Vienna, Austria). Neat serum samples were used and OD was determined at 450 nm (wavelength correction at 550 nm), the limit of detection was 30 pg/mL. All samples were assayed in duplicate. ODs were measured using a FLUOstar® Omega microplate reader (BMG LabTech, Ortenberg, Germany) and concentrations were determined by plotting ODs against known analyte standards on four-parameter logistic curves (GraphPad Prism version 9.5.1, GraphPad Software, Boston, Massachusetts, USA).

#### Post-exercise leg heating intervention

**Fig. 2:**
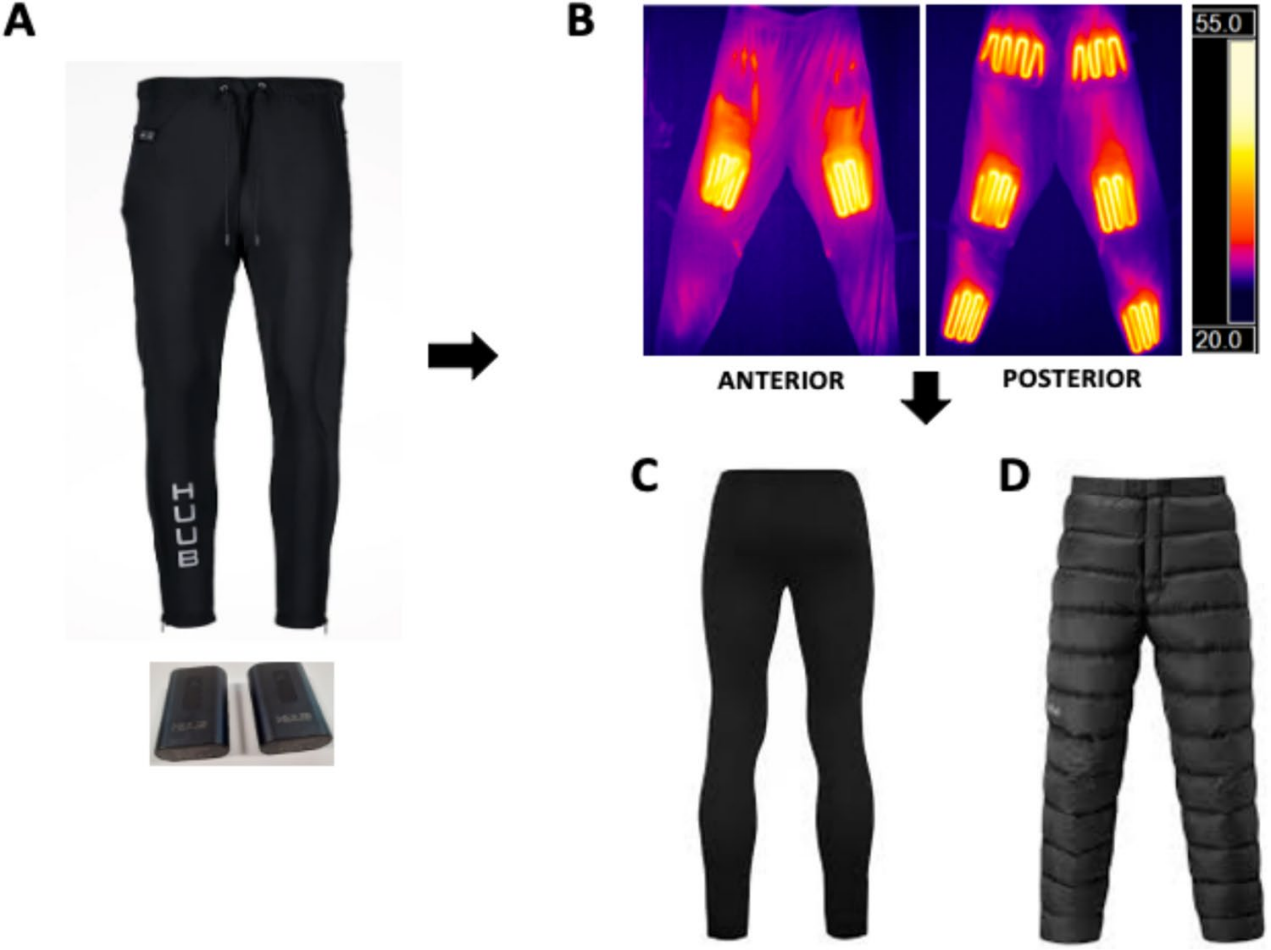
The ensemble passive heating system, which comprised three clothing layers. **A** An electronic lower-limb heating garment (HUUB Design, Derby, England) was worn in immediate contact with the leg skin surface and was set to 43 ℃. The HUUB garments contain electrically heated elements, wired into both sides of the trouser and powered by two 7.4 V lithium batteries that can generate 14.8 W, heating both sides of the garment to 43℃ for a maximum of 2 h. **B** The anatomical sites covered by the HUUB garment were: anterior surface- quadricep muscle region, posterior surface-heating the glutes, hamstrings and calf muscle regions. **C** Elasticated soft-fleeced line thermal leggings (HeatGaurd.™, China, 0.5 Tog 140 denier 90% polyester & 10% elastane) were worn over the HUUB garment to provide thermal insulation and promote a uniform fit of the garment across the skin surface. **D** The outermost thermally insulative layer was a pair of goose down trousers (Naturehike Outdoors, China, 400 tog 20D outer nylon layer, 95% goose down inner fill)

The ensemble passive heating system comprised three clothing layers (Fig. 2). The aim of the layering approach was to better distribute the heat across the lower limbs (see Pilot testing) as the electrical trousers were limited by a panel like design resulting in hot spots. For a typical week, the PAH group were required to complete five heating sessions, of which three had to be post-exercise and two stand-alone heating sessions at any convenient time of the day. For the post-exercise heating sessions, participants were required to wear the heating system (Fig. 2). For 90–120 min immediately (~ 5 min) post-exercise and the duration was kept constant for the stand-alone heating sessions. The limb heating duration was based on previous studies that focused on peripheral heating (Goto et al. 2011; Hafen et al. 2018; Kim et al. 2020), and a range was provided in order to increase participant adherence. It should be noted that participants were allowed to carry on with their normal daily activities indoors whilst wearing the heating system. Additionally, participants were allowed to increase the number of post-exercise heating session as per their discretion.

#### Pilot testing

**Fig. 3:**
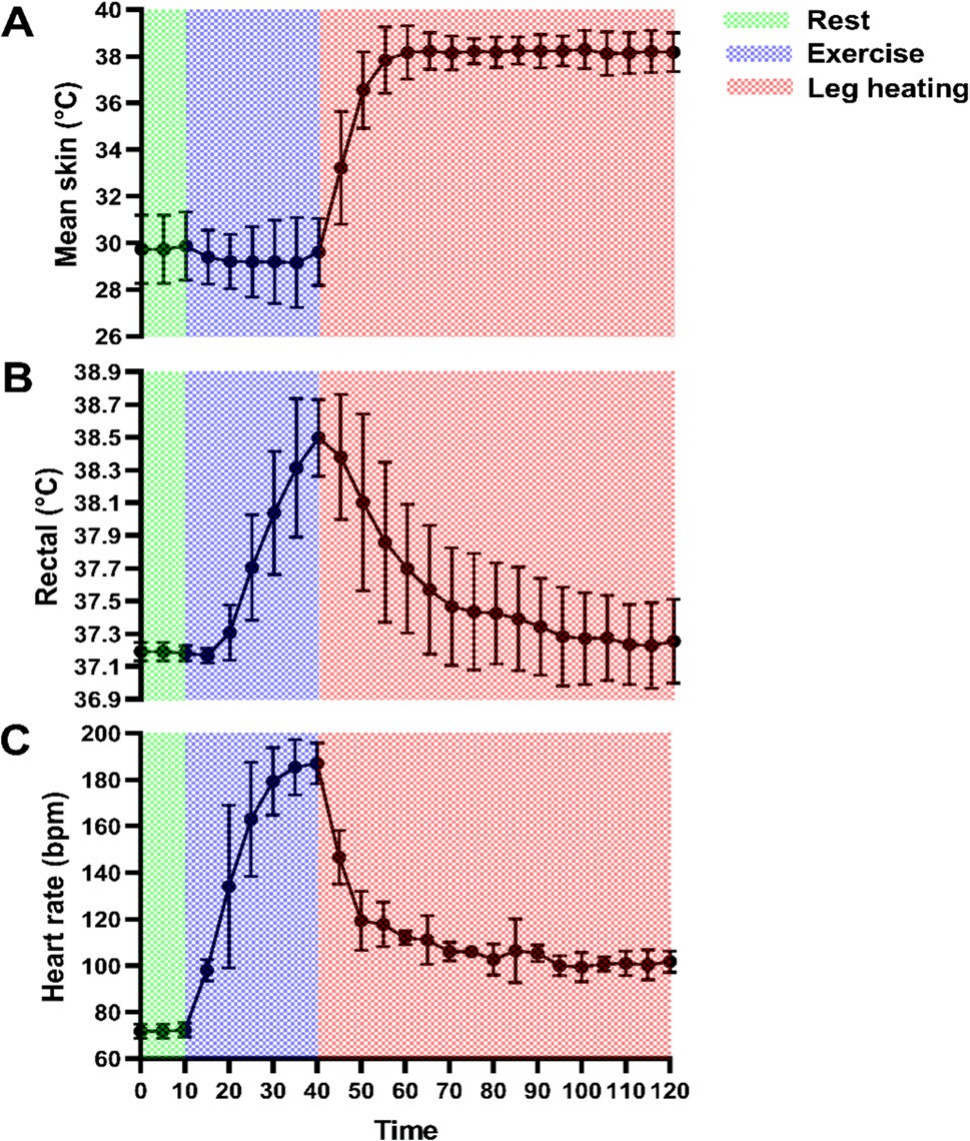
Temporal changes in **A** mean skin (lower leg, avg of six sites), **B** rectal temperature and **C** heart rate during rest (green shaded area, 10 min), exercise (blue shaded area, 30 min) and PH (red shaded area, 80 min) implemented using the ensemble leg heating system

5 (2 females) recreationally trained volunteers took part in a single bout of post-exercise heating. The test was conducted in a thermoneutral chamber (20 °C, 50% RH). Core temperature was monitored using a flexible rectal probe inserted 12 cm past the anal sphincter. Skin thermistors were attached to six sites on the participant’s left side: anterior plane—upper and lower thigh, posterior plane—upper thigh, lower thigh and medial calf and one thermistor attached on the lateral calf. Core and skin temperature were measured throughout testing and data were collected at 5 s intervals (SQ2010; Grant Instruments, Cambridge, United Kingdom). Heart rate was also monitored, and data were collected using Firstbeat data logger (Firstbeat Technologies Oy, Keski-Suomi, Finland). The participant rested for 10 min, after which they began a treadmill run at a self-selected pace (9.0–11.5 km/h) for 30 min. This was immediately followed by 80 min of leg heating using the layered system explained in methods section. During the PH period, the participants wore the ensemble leg heating system and were seated on a chair. Sedentary periods were interrupted by walking intervals of 1 min on a treadmill every 20 min. This mimicked the conditions that would be expected at home or in office settings. The changes in mean skin (average of the six sites), core (rectal) temperature and heart rate during the pilot are presented in Fig. 3.

### Statistical analysis

All statistical analyses were performed using SPSS (Version 28.0.1.1, SPSS Inc., Chicago, Illinois, USA). A two-way repeated measures analysis of co-variance (ANCOVA) was used to determine the main and interaction effects of group (PAH and CON) and time (MID and POST) on all physiological and performance variables. The PRE values were treated as co-variates, with the MID and POST values entered as dependent variables. Training load was analysed using a two-way repeated measures ANOVA (2[group] × 2[time]). Significant main and interaction effects were analysed using Bonferroni-corrected *post hoc* tests. Effect sizes are described in terms of partial eta-squared ($\eta_{p}^{2}$, with $\eta_{p}^{2}$ = 0.01, $\eta_{p}^{2}$ = 0.06 and $\eta_{p}^{2}$ = 0.14 indicating a small, medium and large effect, respectively). Statistical significance was set as *p* $\le$ 0.05. All results are presented as mean ± SD.

## Results

There were no group (*F*_(1,28)_ = 0.176, *p* = 0.678, $\eta_{p}^{2}$ = 0.006) or group by time interaction (*F*_(1,28)_ = 0.234, *p* = 0.632, $\eta_{p}^{2}$ = 0.008) effects for training load during the study. Both groups completed similar number and duration of endurance and resistance training sessions across the 4 weeks (Table 1). The intervention group completed 16 ± 3 post-exercise heating and 5 ± 3 standalone limb heating sessions. The time delay between cessation of exercise and donning the heating system was 5 ± 3 min and the average duration for each PAH session was 111 ± 13 min, indicating the intervention was successfully adhered to.

### Ramp test: $\mathop {\text{V}}\limits^{.}$O_2peak_ and GET

**Fig. 4:**
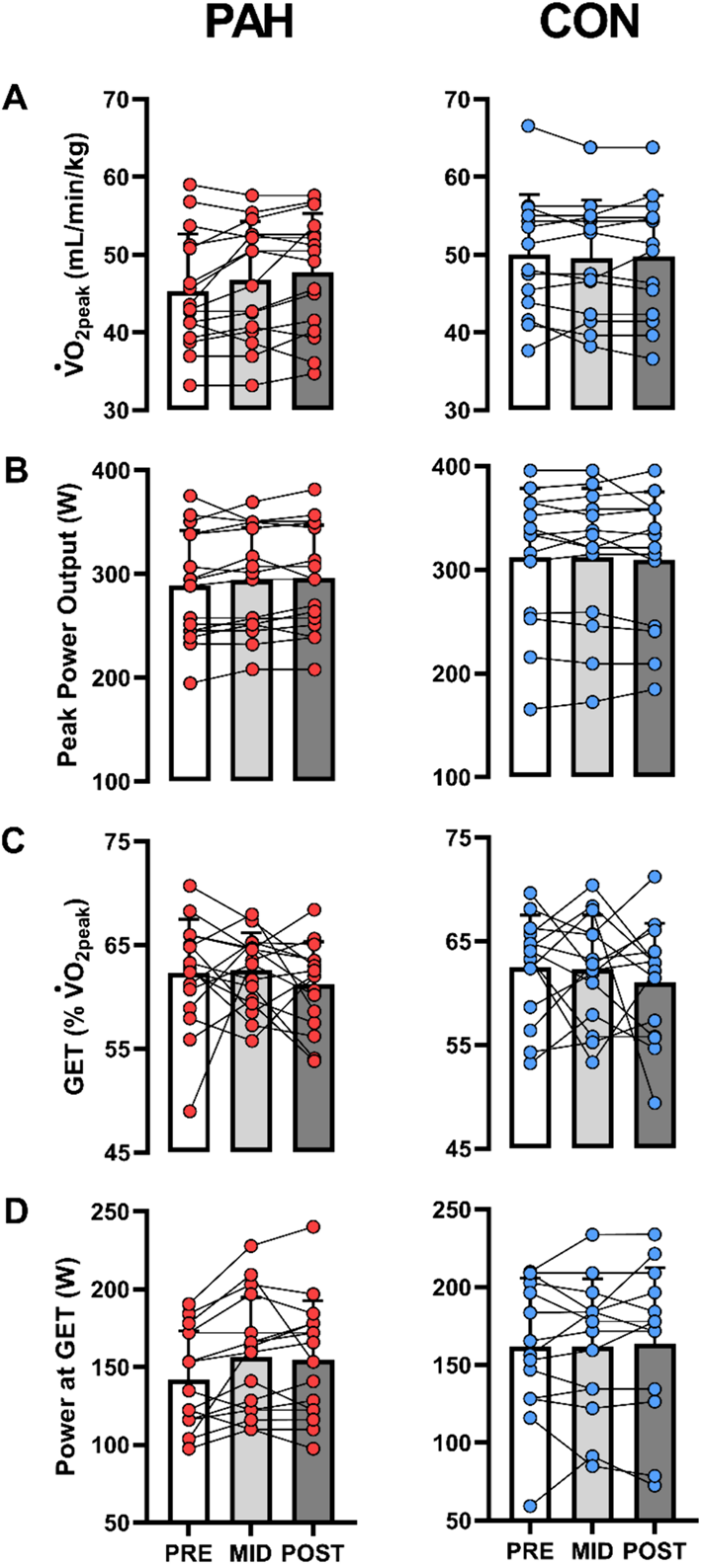
Peak oxygen uptake (**A**), peak power output (**B**), gas exchange threshold (GET) expressed as % of V̇O_2peak_ (**C**) and power output achieved at GET (**D**) derived from ramp test. Bar plots and error bars represent mean ± SD. Circles connected with solid line represent trends for individual participants across PRE, MID and POST for the passive heating (PAH represented by red circles, *n* = 16) and control (CON represented by blue circles, *n* = 14) groups respectively

There were no group (*F*_(1,27)_ = 3.306, *p* = 0.080, $\eta_{p}^{2}$ = 0.109) or group by time interaction effects (*F*_(1,27)_ = 0.878, *p* = 0.357, $\eta_{p}^{2}$ = 0.031) for relative $\mathop {\text{V}}\limits^{.}$O_2peak_. Similarly, there were no group (*F*_(1,27)_ = 2.448, *p* = 0.129, $\eta_{p}^{2}$ = 0.083) or group by time interaction effects (*F*_(1,27)_ = 0.697, *p* = 0.411, $\eta_{p}^{2}$ = 0.025) for peak power output achieved at $\mathop {\text{V}}\limits^{.}$O_2peak_ (Fig. 4).

There were no group (*F*_(1,27)_ = 1.936, *p* = 0.175, $\eta_{p}^{2}$ = 0.067) or group by time interaction effects (*F*_(1,26)_ = 0.081, *p* = 0.778, $\eta_{p}^{2}$ = 0.003) for $\mathop {\text{V}}\limits^{.}$O_2_ at GET. Likewise, there were no group (*F*_(1,27)_ = 0.694, *p* = 0.412, $\eta_{p}^{2}$ = 0.025) or group by time interaction effects (*F*_(1,27)_ = 0.851, *p* = 0.364, $\eta_{p}^{2}$ = 0.031) for GET when expressed as % of V̇O_2peak_. Finally, there were no group (*F*_(1,27)_ = 2.394, *p* = 0.133, $\eta_{p}^{2}$ = 0.081) or group by time interaction effects (*F*_(1,27)_ = 0.405, *p* = 0.530, $\eta_{p}^{2}$ = 0.015) for power achieved at GET (Fig. 4).

### Constant load test: $\mathop {\text{V}}\limits^{.}$˙O_2_ kinetics

There were no group (*F*_(1,27)_ = 2.030, *p* = 0.166, $\eta_{p}^{2}$ = 0.070) or group by time interaction effects (*F*_(1,27)_ = 0.062, *p* = 0.805, $\eta_{p}^{2}$ = 0.002) for $\mathop {\text{V}}\limits^{.}$O_2BASE_ measured prior to the onset of moderate-intensity exercise. There were no group (*F*_(1,27)_ = 2.141, *p* = 0.155, $\eta_{p}^{2}$ = 0.073) or group by time interaction effects (*F*_(1,27)_ = 0.302, *p* = 0.587, $\eta_{p}^{2}$ = 0.011) for the $\mathop {\text{V}}\limits^{.}$O_2AMP_ achieved following the onset of moderate-intensity exercise.

**Fig. 5:**
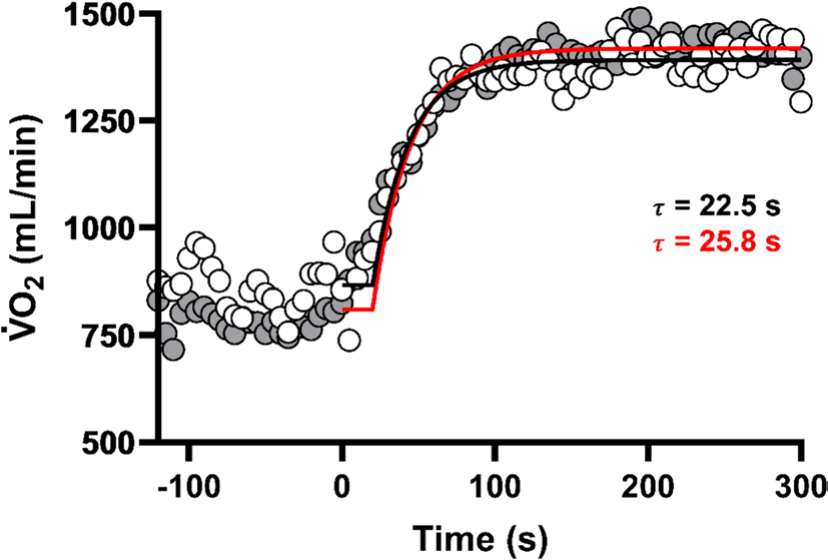
Representative trace for a participant PRE (white circles) and POST (grey circles) PAH. The back and red lines represent the mono-exponential fit (excluding phase I) for PRE and POST, respectively, and depicts an overall slowing down of tau (τ)

There were no group (*F*_(1,27)_ = 0.573, *p* = 0.456, $\eta_{p}^{2}$ = 0.021) but a significant group by time interaction effect for τ measured during moderate-intensity exercise (*F*_(1,27)_ = 4.214, *p* = 0.050, $\eta_{p}^{2}$ = 0.135). Post hoc analysis revealed a slowing of the V̇O_2_ on-kinetics response, denoted by a longer τ (*p* = 0.020) at POST compared to MID in the PAH group (Table 2). Figure 5 displays a representative trace for a participant in PAH. Finally, there were no group (*F*_(1,27)_ = 1.341, *p* = 0.257, $\eta_{p}^{2}$ = 0.047) or group by time interaction effects (*F*_(1,27)_ = 0.569, *p* = 0.457, $\eta_{p}^{2}$ = 0.021) for gross efficiency measured during moderate-intensity exercise (Table 2).

**Table 2:** Parameters derived from the constant work rate test (mean ± SD), for passive heating (*n* = 16) and control (*n* = 14) tested at three time points (PRE, MID and POST)

	Passive heating			Control		
Parameter	Pre	Mid	Post	Pre	Mid	Post
$\mathop V\limits^{.}$O_2BASE_ (mL/min)	901.81 ± 141.06	873.25 ± 136.45	882.68 ± 158.25	908.21 ± 136.21	910.50 ± 151.46	925.71 ± 161.09
$\mathop V\limits^{.}$˙O_2AMP_ (mL/min)	1026.93 ± 284.07	1180.62 ± 338.85	1163.63 ± 348.57	1192.50 ± 404.25	1196.29 ± 380.57	1222.79 ± 459.50
TD (s)	11.26 ± 3.43	11.20 ± 3.17	9.73 ± 3.93	11.86 ± 3.36	12.25 ± 2.34	11.95 ± 3.24
τ (s)^a^	24.95 ± 7.19	24.64 ± 8.31	27.36 ± 6.17^b^	22.96 ± 5.08	23.57 ± 6.22	23.06 ± 6.45
Gross efficiency (%)	19.36 ± 1.47	20.03 ± 1.55	19.84 ± 2.03	20.29 ± 1.71	20.31 ± 1.28	19.91 ± 1.48

$\mathop V\limits^{.}$*O*_*2BASE*_ $\mathop {\text{V}}\limits^{.}$O_2_ during unloaded cycling, $\mathop V\limits^{.}$*O*_*2AMP*_ highest steady-state $\mathop {\text{V}}\limits^{.}$O_2_ achieved above $\mathop {\text{V}}\limits^{.}$O_2BASE_, *TD* time delay, *τ* time constant

^a^Significant interaction effect (*p* = 0.05)

^b^Significantly different between MID and POST in the passive heating group (*p* = 0.020)

### 5-min all-out critical torque test

There were no group (*F*_(1,27)_ = 0.362, *p* = 0.552, $\eta_{p}^{2}$ = 0.013) or group by time interaction effects (*F*_(1,27)_ = 1.680, *p* = 0.206, $\eta_{p}^{2}$ = 0.059) for CT. There were no group (*F*_(1,27)_ = 0.614, *p* = 0.440, $\eta_{p}^{2}$ = 0.022) or group by time interaction effects (*F*_(1,27)_ = 0.055, *p* = 0.817, $\eta_{p}^{2}$ = 0.002) for I′ (Table 3).

**Table 3:** Parameters derived from the 5-min all-out critical torque test in combination with NIRS (mean ± SD). Critical ratio calculated using TSI and HHb signal for passive heating (*n* = *12*) and control (*n* = *9*) group across PRE, MID & POST

	Passive heating			Control		
Parameter	Pre	Mid	Post	Pre	Mid	Post
CT (N.m)	67.92 ± 32.01	73.45 ± 28.98	78.04 ± 32.02	74.23 ± 23.13	78.82 ± 26.09	78.21 ± 21.69
I′ (N.m.s)	4650.9 ± 2030.9	4166.9 ± 2218.1	4101.9 ± 1958.8	4914.8 ± 1944.6	4017.8 ± 1553.1	3825.6 ± 1750.6
CR_T/TSI_ (%N.m/%$${)}$$	0.35 ± 0.09	0.41 ± 0.12	0.46 ± 0.14	0.44 ± 0.08	0.50 ± 0.14	0.54 ± 0.13
τ_1/2_ T/TSI (s)	54.48 ± 20.75	53.63 ± 21.38	61.76 ± 28.01	65.23 ± 27.13	60.17 ± 33.98	54.45 ± 25.98
CR_T/_$\Delta {\text{[HHb]}}$ (%N.m/% a.u.)^a^	2.66 ± 1.73	3.69 ± 2.48	2.78 ± 1.08	2.91 ± 1.57	4.35 ± 4.16	7.09 ± 6.41^bc^
τ_1/2_ T/ $\Delta \left[ {{\text{HHb}}} \right]$ (s)	66.98 ± 21.31	66.25 ± 28.24	70.30 ± 28.17	81.42 ± 36.29	61.75 ± 34.35	61.53 ± 20.46

*CT* critical torque, *I′* impulse prime, *CR*_*T/TSI*_ critical ratio between torque and tissue saturation index (TSI), *τ*_*1/2*_* T/TSI* half-life of torque/TSI curve, *CR*_*T/*_$\Delta {\text{[HHb]}}$ critical ratio between torque and deoxygenation ($\Delta \left[ {{\text{HHb}}} \right])$,*τ*_*1/2*_* T/* $\Delta \left[ {HHb} \right]$ half-life of torque/$\Delta \left[ {{\text{HHb}}} \right]$ curve

^a^Significant interaction effect (*p* = 0.044)

^b^Significant difference between Pre and Mid in Control group (*p* = 0.044)

^c^Significantly different from passive heating at that time point (*p* = 0.031)

### NIRS-derived kinetics of torque/saturation (T/TSI) and deoxygenation ratio (T/HHb)

There were no group (*F*_(1,18)_ = 0.350, *p* = 0.561, $\eta_{p}^{2}$ = 0.019) or group by time interaction effects (*F*_(1,18)_ = 0.274, *p* = 0.607, $\eta_{p}^{2}$ = 0.015) for τ_1/2_ T/TSI derived from T/TSI curve. There were no group (*F*_(1,18)_ = 0.493, *p* = 0.491, $\eta_{p}^{2}$ = 0.027) or group by time interaction effects (*F*_(1,18)_ = 0.033, *p* = 0.858, $\eta_{p}^{2}$ = 0.002) for the critical ratio (CR_T/TSI_) derived from T/TSI curve.

**Fig. 6:**
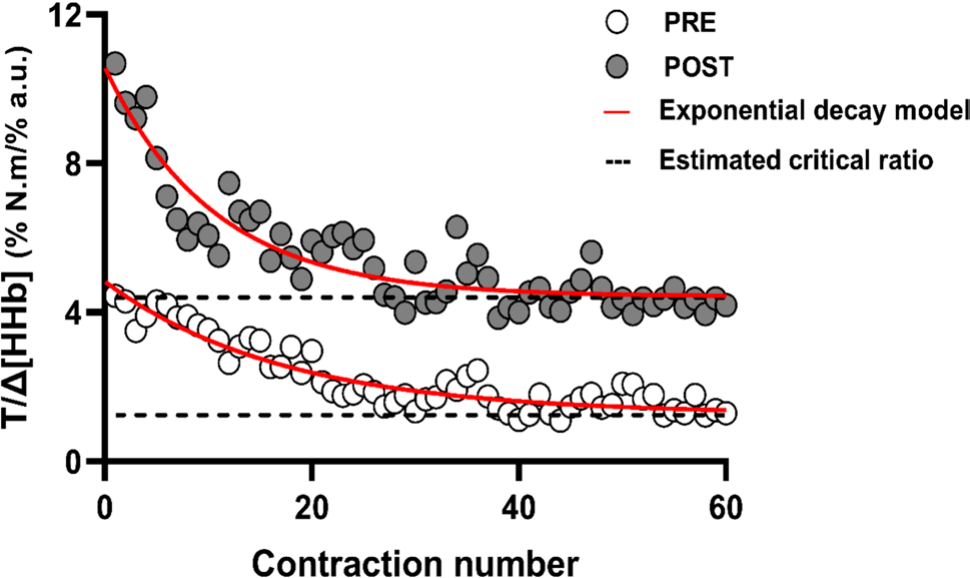
Representative trace for a participant in CON depicting torque (T) and deoxygenation ($\Delta \left[ {{\text{HHb}}} \right]$) ratio of each contraction. The red lines depict the exponential decay fit and the dotted black lines depict model estimated critical ratio (CR_T/_$\Delta {\text{[HHb]}}$) which in this participant improved from PRE to POST

There were no group (*F*_(1,18)_ = 3.656, *p* = 0.072, $\eta_{p}^{2}$ = 0.169) or group by time interaction effects (*F*_(1,18)_ = 0.020, *p* = 0.890, $\eta_{p}^{2}$ = 0.001) for τ_1/2_ T/$\Delta \left[ {{\text{HHb}}} \right]$ derived from T/$\Delta \left[ {{\text{HHb}}} \right]$ curve. There were no group (*F*_(1,18)_ = 2.864, *p* = 0.108, $\eta_{p}^{2}$ = 0.137) but a significant group by time interaction effect (*F*_(1,18)_ = 4.686, *p* = 0.044, $\eta_{p}^{2}$ = 0.206) for CR_T/_$\Delta {\text{[HHb]}}$ derived from T/$\Delta \left[ {{\text{HHb}}} \right]$ curve. Post hoc analysis revealed that CR_T/_$\Delta {\text{[HHb]}}$ was significantly higher (*p* = 0.044) at POST compared to MID within the CON group. Additionally, the CON group had a significantly higher (*p* = 0.031) CR_T/_$\Delta {\text{[HHb]}}$ values at POST compared to PAH (Table 3). Figure 6 presents a representative trace for a participant in CON.

### Flow-mediated dilation

There were no group (*F*_(1,27)_ = 4.138, *p* = 0.052, $\eta_{p}^{2}$ = 0.133) or group by time interaction effects (*F*_(1,27)_ = 1.150, *p* = 0.293, $\eta_{p}^{2}$ = 0.041) for flow-mediated dilation. There was a group effect for baseline arterial diameter (*F*_(1,27)_ = 4.195, *p* = 0.050, $\eta_{p}^{2}$ = 0.134), indicating a larger value for the PAH group compared to CON after adjustment for PRE values; however, there were no group by time interaction effects (*F*_(1,27)_ = 0.682, *p* = 0.416, $\eta_{p}^{2}$ = 0.025). Whilst also under control for PRE values, there was a significant group (*F*_(1,27)_ = 8.266, *p* = 0.008, $\eta_{p}^{2}$ = 0.234) but no group by time interaction effects (*F*_(1,27)_ = 2.122, *p* = 0.157, $\eta_{p}^{2}$ = 0.073) for maximal arterial diameter achieved following occlusion (Table 4).

**Table 4:** Parameters derived from brachial artery flow-mediated dilation test (mean ± SD) for passive heating (*n* = 16) and control (*n* = 14) measured at PRE, MID and POST

	Passive heating			Control		
Parameter	Pre	Mid	Post	Pre	Mid	Post
Flow-mediated dilation (%)	6.46 ± 3.43	6.32 ± 3.57	5.89 ± 1.85	6.08 ± 2.26	5.37 ± 2.10	4.26 ± 1.65
Baseline diameter (mm)#	3.80 ± 0.55	3.86 ± 0.57	3.91 ± 0.56	3.95 ± 0.58	3.93 ± 0.66	3.93 ± 0.66
Max. arterial diameter (mm)#	4.03 ± 0.54	4.09 ± 0.53	4.14 ± 0.56	4.19 ± 0.60	4.14 ± 0.70	4.09 ± 0.65
Time to Max. arterial diameter (s)	35.00 ± 8.10	41.88 ± 13.61	34.75 ± 9.43	33.29 ± 5.90	35.29 ± 6.13	33.50 ± 10.26
Max. vasodilation area (SR_AUC_)	20,305 ± 10,202	19,126 ± 9,059	21,398 ± 10,623	18,666 ± 7,209	18,589 ± 9,595	37,122 ± 78,189

*SR*_*AUC*_ shear rate area under the curve

^a^Significant group effects when controlling for PRE value for baseline diameter (*p* = 0.050) and max. arterial diameter (*p* = 0.008)

There were no group (*F*_(1,27)_ = 1.512, *p* = 0.229, $\eta_{p}^{2}$ = 0.053) and group by time interaction effects (*F*_(1,27)_ = 0.788, *p* = 0.382, $\eta_{p}^{2}$ = 0.028) for time to maximal arterial diameter following occlusion. There were no group (*F*_(1,27)_ = 1.358, *p* = 0.254, $\eta_{p}^{2}$ = 0.048) or group by time interaction effects (*F*_(1,27)_ = 1.554, *p* = 0.223, $\eta_{p}^{2}$ = 0.054) for maximal vasodilation area achieved post-occlusion.

### Serum blood markers

There were no group (*F*_(1,21)_ = 2.187, *p* = 0.154, $\eta_{p}^{2}$ = 0.094) or group by time interaction effects (*F*_(1,21)_ = 3.531, *p* = 0.074, $\eta_{p}^{2}$ = 1.44) for NO (PAH: *n* = 14, CON: *n* = 9). There were no group (*F*_(1,15)_ = 0.577, *p* = 0.459, $\eta_{p}^{2}$ = 0.037) or group by time interaction effects (*F*_(1,15)_ = 1.193, *p* = 0.292, $\eta_{p}^{2}$ = 0.074) for VEGF (PAH: *n* = 11, CON: *n* = 7). Similarly, there were no group or group (*F*_(1,12)_ = 0.013, *p* = 0.912, $\eta_{p}^{2}$ = 0.001) or group by time interaction effects (*F*_(1,12)_ = 0.633, *p* = 0.442, $\eta_{p}^{2}$ = 0.050) for HIF1-α (PAH: *n* = 8, CON: *n* = 7) (Table 5).

**Table 5:** Expression of angiogenic markers in serum acquired from venous blood samples across PRE, MID and POST

	Passive heating			Control		
Parameter	Pre	Mid	Post	Pre	Mid	Post
Nitrate (μmol/L)	27.20 ± 8.41	22.55 ± 7.74	26.66 ± 11.26	21.68 ± 6.93	27.91 ± 9.67	26.09 ± 10.74
VEGF (pg/mL)	141.12 ± 124.71	183.15 ± 187.28	177.22 ± 128.99	165.46 ± 124.55	279.01 ± 224.77	192.70 ± 136.20
HIF1-α (pg/mL $${)}$$	119.00 ± 62.04	117.38 ± 75.03	148.57 ± 76.47	174.44 ± 86.29	197.66 ± 119.91	200.93 ± 146.43

Nitrate (PAH: *n* = 14, CON: *n* = 9)

*VEGF* vascular endothelial growth factor (PAH: *n* = 11, CON: *n* = 7), *HIF1-α* hypoxia inducible factor 1-α (PAH: *n* = 8, CON: *n* = 7)

## Discussion

This study is the first to investigate the effect of chronic (4 week) post-exercise passive leg heating, using a bespoke electrical heating system, on markers of endurance performance in temperate conditions. The primary finding of the study was that a remotely administered intervention in recreationally trained individuals did not have any ergogenic effect, reflected by a lack of change in maximal ($\mathop {\text{V}}\limits^{.}$O_2peak_, peak power output & I′) and sub-maximal components (GET, power at GET, gross efficiency & CT) of endurance performance, compared to exercise only controls. In contrast to our hypotheses, the intervention resulted in an overall slowing of $\mathop {\text{V}}\limits^{.}$O_2_ on-kinetics (τ) during moderate-intensity exercise and based on observations in the CON group, appeared to suppress improvements in the torque/O_2_ extraction ratio (CR_T/_$\Delta {\text{[HHb]}}$) during repeated MVC’s. Additionally, there was no differences in angiogenic blood markers or endothelial function between groups across the study, indicating that PAH conferred no systemic physiological benefits.

### Consideration of the intervention design

The intervention duration and frequency employed in the current study are comparable to previous leg heating studies (Hafen et al. 2018; Kim et al. 2019, 2020); however, most of the leg heating sessions in the current study were completed post-exercise (~ 16 days) as opposed to stand-alone leg heating sessions (~ 5 days). Accordingly, consecutively administering two separate physiological stressors within the given timeframe may not have been optimal for the level of athletes (recreationally trained) recruited herein. Pilot data for the current study demonstrated that self-paced running (30 min) increased core temperature by ~ 1 °C. This is equivalent to the elevations reported during sub-maximal (65% $\mathop {\text{V}}\limits^{.}$O_2peak_) running in temperate conditions (Zurawlew et al. 2016). Donning the ensemble heating system immediately post-exercise raised mean skin temperature by ~ 8 °C, with core temperature recovering to baseline values within an hour and heart rate being elevated by ~ 30 beats/min relative to baseline for the entirety of the passive heating period (Fig. 3). It could be argued that the observed elevation in core temperature is related to the ergogenic effect of passive heating interventions (Dalleck et al. 2019; Kirby et al. 2021; Scoon et al. 2007). Although, increases in skeletal muscle capillarisation and aerobic capacity have been reported with only skin heating interventions that do not raise core temperature (Hesketh et al. 2019). Alternatively, the rise in muscle temperature during passive heating may be of greater importance than previously considered, especially for induction of peripheral oxidative adaptations (Heinonen et al. 2011; Marchant et al. 2023). Previous work by Raccuglia et al. (2016) has shown that lower limb heating using a water perfused suit increased quadriceps skin temperature to ~ 38 °C, subsequently delaying the decrease in deep-muscle temperature and raising mid- and superficial-muscle temperature 30 min post warm-up. The heating system used herein was equally effective in raising mean leg skin temperature to 38.2 ± 0.1 °C (Fig. 3), which is greater than that reported previously, using a similar electrically heated trouser (Faulkner et al. 2013; Raccuglia et al. 2016) and the superiority can be attributed to the addition of the insulative layers (Fig. 2). Although we did not directly measure muscle temperature, it is highly likely that the micro-climate created with the current system could have had a similar influence on muscle temperature kinetics post-exercise (Faulkner et al. 2013; Raccuglia et al. 2016). Consequently, the prolonged periods of homeostatic disturbance in the post-exercise period (Cheng et al. 2021; Francisco et al. 2021) might have acutely delayed the development of beneficial adaptations associated with leg heating (Hafen et al. 2018; Kim et al. 2020).

### Potential maladaptive effect of post-exercise passive leg heating

Our study demonstrated an overall slowing of $\mathop {\text{V}}\limits^{.}$O_2_ kinetics after four weeks of PAH. The τ represents time required to achieve steady-state from rest-to-exercise and is considered to be a reliable, non-invasive estimation of muscle $\mathop {\text{V}}\limits^{.}$O_2_ kinetics (Rossiter et al. 1999). In healthy individuals tested in normoxic conditions, overcoming the inertia of activating oxidative phosphorylation is considered to be the primary determinant of τ, as opposed to the delivery/perfusion of O_2_ across the musculature (Poole and Jones 2012). Thus, our results are indicative of a maladaptive response at the mitochondrial level. It is difficult to fully explain this response, as direct molecular mechanisms were not measured. A potential explanation could be that the chronic nature of the current study may have contributed to redox imbalance at the peripheral level (King et al. 2016; Slimen et al. 2014). This is supported by findings from *in vitro* models, where hyperthermia-induced reactive oxygen species impairs the transfer of energy across the electron transport chain and reduces oxidative phosphorylation efficiency (Davis et al. 2023; Zhou et al. 2020). However, future studies are required to confirm this *in-vivo*, as previous work by Koga et al. (1997) reported no deleterious effect of acute leg heating on τ during moderate-intensity exercise. However, it should be noted that the exercise intensity for the constant work rate protocol was adjusted for changes in fitness across PRE, MID and POST (Fig. 4D; PAH: $\sim$141, $\sim$156 & $\sim$154 W vs. CON: $\sim$161, $\sim$161 & $\sim$163 W, respectively). It was deemed appropriate to take this approach to ensure methodological rigour, such that all participants were compared at the same physiological domain across the 4-week period. Accordingly, we noticed a non-significant increase in $\mathop {\text{V}}\limits^{.}$O_2AMP_ as a result of an increased work rate from PRE to POST (Table 2); however, the $\sim$13 W increase in power at GET remained in the moderate domain and would not affect τ, based on previous reports that have demonstrated no change in τ when exercising in close proximity to the GET (Keir et al. 2015).

Another novel approach in the current study was the simultaneous measurement of peripheral O_2_ kinetics using NIRS during the 5 min AOCT test. Similar to gross efficiency, this reflects the ratio between mechanical work done and the metabolic cost incurred (Coyle et al. 1992), CR_T/TSI_ and CR ${\text{T/}}\Delta {\text{[HHb]}}$ depicts the ratio of CT to quadricep O_2_ availability or extraction, respectively. Interestingly, a progressive improvement in CR ${\text{T/}}\Delta {\text{[HHb]}}$ was noted in the CON group, with no changes CR_T/TSI_ in either group. The $\Delta \left[ {{\text{HHb}}} \right]$ signal derived from NIRS is blood flow insensitive and is used to estimate O_2_ extraction at the micro-circulatory level during muscular contractions (De Blasi et al. 1994). A higher CR ${\text{T/}}\Delta {\text{[HHb]}}$ ratio, therefore, indicates that the CON group became more efficient (i.e. lesser O_2_ extraction) in achieving CT compared to PAH group. The improvements in the CON group could be attributed to a learning effect or improvements in skeletal muscle O_2_ metabolism reported with routine endurance training. In comparison to all other laboratory tests conducted in this study, the 5 min AOCT was the most unfamiliar/novel test and, therefore, a learning effect (PRE, MID and POST) may have occurred. To control for this, a familiarisation visit was conducted before PRE and both groups had similar experience at baseline. Alternatively, improved adherence to training after inclusion into the study (due to being involved in the study and daily logging) might have facilitated beneficial adaptations. However, training load was similar between groups across the study, which might indicate a suppression of similar training adaptions in the PAH group.

It is noteworthy that two sub-maximal parameters of endurance performance, determined via two separate tests (τ & CR ${\text{T/}}\Delta {\text{[HHb]}}$), which are primarily linked to mitochondrial function (De Blasi et al. 1994; Poole and Jones 2012), displayed a dampened-like response to the PAH intervention. A comparison of current findings with the existing literature on leg heating is complex due to our interventional approach being non-conventional (post-exercise and remotely administered). Keeping this in mind, we speculate that the maladaptive response observed in PAH may share similarities with functional over-reaching (usually 3-to-4 weeks in duration), commonly used to elicit endurance performance improvements (Aubry et al. 2014; Coutts et al. 2006; Le Meur et al. 2013). Here, both intensified training (Halson et al. 2003; Robson-Ansley et al. 2007) and leg heating (Hoekstra et al. 2021; Kuhlenhoelter et al. 2016) have shown to transiently evoke an inflammatory response, which is a necessary element of adaptation (Kuennen et al. 2011; Paulsen et al. 2014), but also indirectly indicates a commonality in the physiological stress induced by the separate stimuli. Indeed, this may also explain why aerobic capacity or performance super-compensates following sufficient recovery, typically 1–2 weeks after over-reaching (Aubry et al. 2014; Coutts et al. 2006; Le Meur et al. 2013) and 4 days after heat acclimation (Peel et al. 2023; Waldron et al. 2019). Additionally, the only study, to date, using electrically heated garments during exercise with contralateral-limb control (Maunder et al. 2024), reported improvements in oxidative phosphorylation (complex I and II) enzyme activity. Notably, the tissue biopsy samples were taken 4–6 days post the last day of intervention (Maunder et al. 2024), rather than the 24 h of the current study. Extrapolating these trends in adaptations, and in accordance with general adaptation syndrome theory (Selye 1950), the 24 h recovery period provided in the current study might have not been adequate to resolve any cumulative fatigue in PAH. This also provides a reasonable explanation for peripheral maladaptation, especially as heating was restricted to, and directly applied over, the legs.

### Parameters of endothelial function and vascular structure

Brachial artery dilation, as assessed via flow-mediated dilation test, did not differ between group across the intervention. This response is primarily dependent on the ability of the vessel’s endothelial layer to release NO (Celermajer et al. 1992; Green et al. 2014). The shear-stimulus is considered to be the major driver for enhancing endothelial-mediated release of NO (Naylor et al. 2011; Tinken et al. 2010) and promoting arterial remodelling (Green et al. 2004). It is well documented that whole-body and lower limb skin heating can increase blood flow and shear rate in conduit vessels (Chiesa et al. 2016) and, therefore, we sought to investigate whether leg heating could improve upstream vessel function or structure as seen in lower-limb exercise interventions (Birk et al. 2012). The lack of change demonstrated in vascular parameters measured in the current study contradicts earlier findings, where an improvement in brachial artery function was reported as a result of lower body (Carter et al. 2014) and whole-body hot-water immersion (Brunt et al. 2016) or sauna bathing (Imamura et al. 2001). These contrasting findings may point towards the importance of elevating core temperature as an essential stimulus for enhancing vascular function (Brunt et al. 2016; Carter et al. 2014; Imamura et al. 2001). Notwithstanding the lack of change in artery function, there were group effects, demonstrating a larger baseline and maximal vasodilatory arterial diameter in the PAH group compared to CON after accounting for baseline values (PRE), thus indicating a change in vessel size during rest and in the hyperaemic state. These measures have been proposed to represent arterial remodelling and potentially explain the biphasic return of flow mediated dilation scores back to baseline with chronic heating (Carter et al. 2014) or exercise interventions (Naylor et al. 2011; Tinken et al. 2010). However, this trend was not found in the current study and, coupled with a lack of change in angiogenic serum biomarkers, caution is warranted when interpreting these group effects.

## Limitations

This study has some limitations. Firstly, as the study aimed to assess a remotely applied post-exercise leg heating intervention, the endurance exercise conducted by participants was self-selected (participants continued their routine training) and unsupervised. As such, participants were not restricted to a specific endurance exercise type, mode or intensity, which may have added a source of variance in the current study. The session RPE method (Foster et al. 2001) was utilised to monitor training. More objective methods, such as heart rate monitors or inertial sensors, might have increased the precision of training load quantification; however, the session RPE is a reliable and valid method for estimating internal training load across various training types (Haddad et al. 2017), yielding a singular training load metric that would be appropriate for making comparisons across training regimes and attempting to balance across groups. Secondly, the current study was only able to recruit five female participants and, therefore, the findings may not be adequately transferable to this population. Furthermore, hormonal fluctuations occurring across the menstrual cycle were not controlled for in the current study. We are uncertain whether this would have a major effect on the study outcomes, given that the menstrual phase does not appear to influence $\mathop {\text{V}}\limits^{.}$˙O_2peak,_ physiological thresholds (Ekberg et al. 2023) or $\mathop {\text{V}}\limits^{.}$˙O_2_ kinetics (Mattu et al. 2020). Lastly, we assessed circulating serum nitric oxide metabolites as a proxy for eNOS activity; however, the technique used herein (using serum) limited our analysis to quantification of nitrate concentration. This is an important consideration as serum nitrate is affected by various eNOS independent factors (Kelm 1999), which limit its capacity to reflect changes in NO, leaving questions over the role of NO in the reported outcomes of the present study.

## Conclusion

In conclusion, the current remotely administered post-exercise passive leg heating intervention did not improve hallmark endurance characteristics or systemic markers of adaptation but, rather, had a negative effect on some sub-maximal exercise parameters. Therefore, further studies are required to investigate the importance of key intervention variables (i.e. training status, heating intensity, volume and recovery) when utilising similar mobile leg heating systems, with the intention of augmenting adaptations associated with endurance training.

## Supplementary Information

Below is the link to the electronic supplementary material.

Supplementary file1 (DOCX 34 KB) Supplementary file- Details the pilot testing data (Description, Figure S1 and Figure S2) and provides mean ± SD for blood markers (Table S1).

## Data Availability

Data will be made available upon reasonable request.

## Abbreviations

$\mathop {\text{V}}\limits^{.}$CO_2_: Carbon-dioxide production
$$\mathop {\text{V}}\limits^{.}$$O_2_: Oxygen uptake
$$\mathop {\text{V}}\limits^{.}$$O_2AMP_: $$\mathop {\text{V}}\limits^{.}$$O_2_ amplitude
$$\mathop {\text{V}}\limits^{.}$$O_2BASE_: $$\mathop {\text{V}}\limits^{.}$$O_2_ baseline
$\mathop {\text{V}}\limits^{.}$O_2_ kinetics: Pulmonary oxygen uptake kinetics
$\mathop {\text{V}}\limits^{.}$O_2peak_: Peak oxygen uptake
5 min AOCT: Five-minute all out critical torque test
ANCOVA: Analysis of co-variance
CON: Control group
CR_T/__Δ[HHb]_: Critical torque/deoxygenated haemoglobin ratio
CR_T/TSI_: Critical torque/tissue saturation index ratio
CT: Critical torque
D: Arterial diameter
D_BASELINE_: Baseline arterial diameter
D_MAX_: Maximal arterial diameter
eNOS: Endothelial nitric oxide synthase
GET: Gas exchange threshold
HHb: Deoxygenated haemoglobin
HIF-1a: Hypoxia-inducible factor 1-alpha
I: Total impulse
I′: Impulse prime
MVC: Maximal voluntary contraction
NIRS: Near-infrared spectroscopy
PAH: Post-exercise passive leg heating group
RER: Respiratory exchange ratio
RPE: Rate of perceived exertion
SD: Standard deviation
SR: Sheer rate
SR_AUC_: Sheer rate area under the curve
TD: Time delay
TL: Training load
TSI: Tissue saturation index
V: Mean velocity
$$\mathop {\text{V}}\limits^{.}$$E: Ventilatory equivalent
VEGF: Vascular endothelial growth factor
W: Watts
τ (tau): Time constant
