# Supplementary material for: The effect of a 4-week, remotely administered, post-exercise passive leg heating intervention on determinants of endurance performance
Source: Eur J Appl Physiol. 2024 Jul 25;124(12):3631–47. doi: 10.1007/s00421-024-05558-4 (PMC11569002; doi:10.1007/s00421-024-05558-4)
Supplement: Supplementary file 1 — Supplementary file1 (DOCX 34 KB) Supplementary file- Details the pilot testing data (Description, Figure S1 and Figure S2) and provides mean ± SD for blood markers (Table S1). [file 421_2024_5558_MOESM1_ESM.docx]

Covariate adjusted data for **Figure 4**.

**V̇O_2peak_ (mL/min/kg)**

Group effect-

| Group | Mean |
| --- | --- |
| PAH | 49.30 ± 2.83 |
| CON | 47.37 ± 2.84 |

Group*time interaction effect-

| Group | MID | POST |
| --- | --- | --- |
| PAH | 48.83 ± 2.60 | 49.78 ± 3.40 |
| CON | 47.28 ± 2.61 | 47.47 ± 3.41 |

**Peak power output (W)**

Group effect-

| Group | Mean |
| --- | --- |
| PAH | 305.36 ± 10.72 |
| CON | 299.16 ± 10.73 |

Group*time interaction effect-

| Group | MID | POST |
| --- | --- | --- |
| PAH | 304.42 ± 10.91 | 306.32 ± 13.79 |
| CON | 300.15 ± 10.92 | 298.17 ± 13.81 |

**Gas exchange threshold (%)**

Group effect-

| Group | Mean |
| --- | --- |
| PAH | 61.88 ± 7.22 |
| CON | 59.67 ± 7.22 |

Group*time interaction effect-

| Group | MID | POST |
| --- | --- | --- |
| PAH | 62.58 ± 4.13 | 61.17 ± 12.40 |
| CON | 62.33 ± 4.13 | 57.03 ± 12.39 |

**Power output achieved at GET (W)**

Group effect-

| Group | Mean |
| --- | --- |
| PAH | 164.57 ± 20.60 |
| CON | 152.69 ± 20.65 |

Group*time interaction effect-

| Group | MID | POST |
| --- | --- | --- |
| PAH | 165.70 ± 18.90 | 163.45 ± 25.06 |
| CON | 151.85 ± 18.95 | 153.53 ± 25.11 |

Covariate adjusted data for **Table 2**.

**V̇O_2BASE_ (mL/min)**

Group effect-

| Group | Mean |
| --- | --- |
| PAH | 880.86 ± 65.06 |
| CON | 914.79 ± 65.06 |

Group*time interaction effect-

| Group | MID | POST |
| --- | --- | --- |
| PAH | 876.04 ± 63.01 | 885.69 ± 78.68 |
| CON | 907.31 ± 63.01 | 922.28 ± 78.68 |

**V̇O_2AMP_ (mL/min)**

PRE covariate adjusted value = 1104.2000

Group effect-

| Group | Mean |
| --- | --- |
| PAH | 1242.56 ± 208.70 |
| CON | 1129.04 ± 209.11 |

Group*time interaction effect-

| Group | MID | POST |
| --- | --- | --- |
| PAH | 1248.55 ± 197.90 | 1236.56 ± 246.51 |
| CON | 1118.66 ± 198.29 | 1139.42 ± 247.00 |

**𝜏 (s)**

Group effect-

| Group | Mean |
| --- | --- |
| PAH | 25.43 ± 5.01 |
| CON | 24.03 ± 5.02 |

Group*time interaction effect-

| Group | MID | POST |
| --- | --- | --- |
| PAH | 23.96 ± 5.92 | 26.91 ± 5.15 |
| CON | 24.36 ± 5.93 | 23.71 ± 5.15 |

Covariate adjusted data for **Table 3.**

**CT (N.m)**

Group effect-

| Group | Mean |
| --- | --- |
| PAH | 78.39 ± 10.60 |
| CON | 75.48 ± 10.61 |

Group*time interaction effect-

| Group | MID | POST |
| --- | --- | --- |
| PAH | 76.07 ± 11.92 | 80.72 ± 12.19 |
| CON | 75.82 ± 11.93 | 75.14 ± 12.19 |

**I’ (N.m.s)**

Group effect-

| Group | Mean |
| --- | --- |
| PAH | 4209.7 ± 1302.1 |
| CON | 3835.7 ± 1302.3 |

Group*time interaction effect-

| Group | MID | POST |
| --- | --- | --- |
| PAH | 4242.8 ± 1529.1 | 4176.5 ± 1450.2 |
| CON | 3931.1 ± 1529.4 | 3740.4 ± 1450.4 |

**CR_T/TSI_ (%N.m/% a.u.**$\text{)}$

Group effect-

| Group | Mean |
| --- | --- |
| PAH | 0.45 ± 0.10 |
| CON | 0.49 ± 0.08 |

Group*time interaction effect-

| Group | MID | POST |
| --- | --- | --- |
| PAH | 0.44 ± 0.12 | 0.47 ± 0.14 |
| CON | 0.46 ± 0.12 | 0.52 ± 0.14 |

**𝜏_1/2_T/TSI (s)**

Group effect-

| Group | Mean |
| --- | --- |
| PAH | 59.75 ± 19.60 |
| CON | 54.56 ± 19.67 |

Group*time interaction effect-

| Group | MID | POST |
| --- | --- | --- |
| PAH | 57.09 ± 21.68 | 62.42 ± 28.03 |
| CON | 55.56 ± 21.77 | 53.57 ± 28.14 |

**CR_T/_**$\text{∆[}\text{HHb}\text{]}$ **(%N.m/% a.u.)**

Group effect-

| Group | Mean |
| --- | --- |
| PAH | 3.34 ± 3.02 |
| CON | 5.59 ± 3.02 |

Group*time interaction effect-

| Group | MID | POST |
| --- | --- | --- |
| PAH | 3.77 ± 3.14 | 2.90 ± 3.91 |
| CON | 4.25 ± 3.14 | 6.94 ± 3.91 |

**𝜏_1/2_T/** $\boldsymbol{\Delta}\mathbf{[HHb]}$ **(s)**

Group effect-

| Group | Mean |
| --- | --- |
| PAH | 71.89 ± 17.48 |
| CON | 56.85 ± 17.66 |

Group*time interaction effect-

| Group | MID | POST |
| --- | --- | --- |
| PAH | 70.33 ± 25.63 | 73.41 ± 21.60 |
| CON | 56.32 ± 25.75 | 57.38 ± 21.70 |

Covariate adjusted data for **Table 4.**

**Flow mediated dilation (%)**

Group effect-

| Group | Mean |
| --- | --- |
| PAH | 6.01 ± 1.47 |
| CON | 4.92 ± 1.47 |

Group*time interaction effect-

| Group | MID | POST |
| --- | --- | --- |
| PAH | 6.18 ± 2.06 | 5.84 ± 1.58 |
| CON | 5.52 ± 2.06 | 4.32 ± 1.58 |

**Baseline diameter (mm)**

Group effect-

| Group | Mean |
| --- | --- |
| PAH | 3.96 ± 0.15 |
| CON | 3.85 ± 0.15 |

Group*time interaction effect-

| Group | MID | POST |
| --- | --- | --- |
| PAH | 3.93 ± 0.14 | 3.98 ± 0.19 |
| CON | 3.85 ± 0.14 | 3.85 ± 0.19 |

**Max. arterial diameter (mm)**

Group effect-

| Group | Mean |
| --- | --- |
| PAH | 4.18 ± 0.14 |
| CON | 4.03 ± 0.15 |

Group*time interaction effect-

| Group | MID | POST |
| --- | --- | --- |
| PAH | 4.16 ± 0.15 | 4.21 ± 0.18 |
| CON | 4.05 ± 0.15 | 4.01 ± 0.19 |

**Time to Max. arterial diameter (s)**

Group effect-

| Group | Mean |
| --- | --- |
| PAH | 37.90 ± 6.74 |
| CON | 34.86 ± 6.74 |

Group*time interaction effect-

| Group | MID | POST |
| --- | --- | --- |
| PAH | 41.29 ± 9.66 | 34.52 ± 9.81 |
| CON | 35.95 ± 9.67 | 33.76 ± 9.82 |

**Max. vasodilation area**

Group effect-

| Group | Mean |
| --- | --- |
| PAH | 18820 ± 24980 |
| CON | 29498 ± 24886 |

Group*time interaction effect-

| Group | MID | POST |
| --- | --- | --- |
| PAH | 18660 ± 7712 | 18980 ± 46764 |
| CON | 19120 ± 7715 | 39875 ± 46778 |

Covariate adjusted data for **Table 5**.

**Nitrate (μmol/L)**

Group effect-

| Group | Mean |
| --- | --- |
| PAH | 23.28 ± 8.27 |
| CON | 29.06 ± 8.38 |

Group*time interaction effect-

| Group | MID | POST |
| --- | --- | --- |
| PAH | 21.52 ± 8.31 | 25.04 ± 9.61 |
| CON | 29.51 ± 8.42 | 28.62 ± 9.74 |

**VEGF (pg/mL)**

Group effect-

| Group | Mean |
| --- | --- |
| PAH | 190.51 ± 79.05 |
| CON | 219.62 ± 79.13 |

Group*time interaction effect-

| Group | MID | POST |
| --- | --- | --- |
| PAH | 195.31 ± 127.65 | 185.72 ± 71.93 |
| CON | 259.89 ± 126.80 | 179.35 ± 72.01 |

**HIF1- α (pg/mL)**

Group effect-

| Group | Mean |
| --- | --- |
| PAH | 164.90 ± 48.68 |
| CON | 162.81 ± 48.91 |

Group*time interaction effect-

| Group | MID | POST |
| --- | --- | --- |
| PAH | 146.47 ± 77.13 | 183.33 ± 84.99 |
| CON | 164.41 ± 79.70 | 161.20 ± 85.41 |
